# Opto-electric investigation for Si/organic heterojunction single-nanowire solar cells

**DOI:** 10.1038/s41598-017-15300-0

**Published:** 2017-11-06

**Authors:** Zhenhai Yang, Zhaolang Liu, Jiang Sheng, Wei Guo, Yuheng Zeng, Pingqi Gao, Jichun Ye

**Affiliations:** 1 Ningbo Institute of Material Technology and Engineering, Chinese Academy of Sciences, Ningbo, 315201 China; 2 School of Materials Science and Engineering, Shanghai University, Shanghai, 200072 China

## Abstract

Recently, silicon single nanowire solar cells (SNSCs) serving as the sustainable self-power sources have been integrated into optoelectronic nanodevices under the driver of technology and economy. However, conventional SNSC cannot provide the minimum energy consumption for the operation of nanodevices due to its low power conversion efficiency (PCE). Here, we propose an innovative approach to combine the *n*-type silicon nanowires (SiNWs) with *p*-type poly(3,4-ethylthiophene):poly(styrenesulfonate) (PEDOT:PSS) to form the *p* ^+^ *n* heterojunction, which shows superior opto-electric performances. Besides, PEDOT:PSS also acts as a natural anti-reflection coating (ARC) with an excellent light-trapping capability, especially in the short-wavelength range. Importantly, the photovoltaic performances of Si/PEDOT:PSS SNSC can be well maintained even in large surface recombination velocity, due to the efficient field-effect passivation of PEDOT:PSS. The minority carrier concentration at outer surface of shallow *p* ^+^ *n* heterojunction is greatly reduced by the electric field, drastically suppressing the surface recombination compared to the conventional *p-i-n* homojunction SNSC. Furthermore, larger junction area of *p* ^+^ *n* heterojunction facilitates the separation of photo-generated charge carriers. These results demonstrate that the Si/PEDOT:PSS SNSC is a promising alternative for micro power application.

## Introduction

Compared to conventional silicon wafers, silicon nanowires (SiNWs) have emerged as an attractive alternative for low-cost and high-efficiency solar cells because of their unique geometrical and photoelectric features, such as: superior light-harvesting capability^1–4^ and efficient charge carriers collection (potential utilization of lower-quality Si materials, e.g., upgraded metallurgical-grade silicon^5^). Recently, the applications of single nanowire solar cells (SNSCs) have been attracted much attention, which serve as the integrated electric source to power nanologics, nanodiodes, nanophotodetectors, and nanosensors^6–9^. For example, Tian *et al*. reported the coaxial SNSC consisted of *p*-*i*-*n* doped configuration, yielding a PCE of 200 pW per SNSC as the power source for nanologic^7^. However, the inferior PCE of SNSC prevent it from contending competitive photovoltaic technology and relevant applications. In order to boost the PCE, a number of strategies have been implemented. Li *et al*. designed the asymmetrical crescent nanostructures^10^ and off-axial SiO_2_ coating layers^11^ to dramatically improve the light-harvesting of SNSC. Decorating Ag plasmons^12^ or adding Ag reflective substrate^13^ is also a feasible method to enhance the light absorption. Nevertheless, there is still a large space to improve the PCE of SNSC for more practical application.

However, the conventional fabrication techniques for coaxial *p*-*i*-*n* doped SNSC are very complicated: *p*-type cores are realized by vapor-liquid-solid technology with the aid of gold catalyst, intrinsic layers are grown by vapor-solid growth, and *n*-type shells are achieved by chemical vapor deposition followed by rapid thermal annealing at about 860 °C^7,14,15^. This complicated procedures not only increase the manufacture cost, but also result in a large number of surface defects. Moreover, the perfect homojunction SNSC is hardly achieved as a basis of this *p*-*i*-*n* type, because SiNWs will be oxidized or destroyed at high temperature during the phosphorous (boron) diffusion processes, which intrinsically restricts the photoelectrical properties of SNSC^16–18^. Recently, the silicon/organic heterojunction consisted of dopant-free *p*-type polymer PEDOT:PSS and *n*-type crystalline silicon is adequately utilized to realize the high performances with PCE up to 20.6%^19–22^. Many strategies are adopted to improve the PCE of Si/PEDOT:PSS heterojunction solar cells (HSCs), including the passivation layer^19,23,24^, silicon surface microstructures^25–27^, interface modification^20,21^ and antireflection layers^28,29^. Back PEDOT:PSS/Si HSCs have got the exceeding 20% PCE^30^, while front PEDOT:PSS/Si HSCs have received more 16% PCE^31^. Especially, for SNSCs, PEDOT:PSS can be easily wrapped on the surface of SiNWs to form the conformal device by the vapor phase polymerization^32^ or spin-coating method^33^ at low temperature. Importantly, a strong inversion layer at the Si/PEDOT:PSS interface from the difference of Fermi level of Si (4.2 eV at *P* doping concentration of 10^17^ cm^−3^) and work function of PEDOT:PSS (5.0–5.1 eV) efficiently prevent the electrons diffusing into *p*-type PEDOT:PSS layer and thus suppress the surface carrier recombination. Moreover, the Si/PEDOT:PSS core/shell nanowire arrays have been successful fabricated and applied in Si/PEDOT:PSS HSC with a relatively high PCE^24,26,34,35^, providing the possibility of achieving high performance Si/PEDOT:PSS SNSCs. Therefore, based on these superior features, it is a simple and efficient way to implement the coaxial Si/PEDOT:PSS SNSC, as a potential alternative for high efficiency single nanowire device. It is deserved to thoroughly investigate the optical and electrical properties of Si/PEDOT:PSS SNSC.

In this study, we systematically evaluate the photoelectrical performances of Si/PEDOT:PSS SNSC by implementing numerical simulation. PEDOT:PSS layer displays the excellent light-trapping ability due to its antireflection property and nano-focusing effect. More importantly, the photovoltaic performance of Si/PEDOT:PSS SNSC including the short circuit current density (*J*_sc_) and open circuit voltage (*V*_oc_) can be well maintained with increasing the surface recombination velocity. The minority carrier and potential distribution are investigated in details, which are intrinsically contributed to this phenomenon. Additionally, an Ag reflector is introduced to the rear side of SNSC to further improve the light harvesting. Thus, an excellent PCE of 8.68% for Si/PEDOT:PSS SNSC can be expected, which is much higher than that of homojunction systems (5.02% for bare and 7.80% for Si_3_N_4_ SNSCs), with an outstanding enhancement of 72.91% and 11.13%, respectively.

## Results

**Figure 1:**
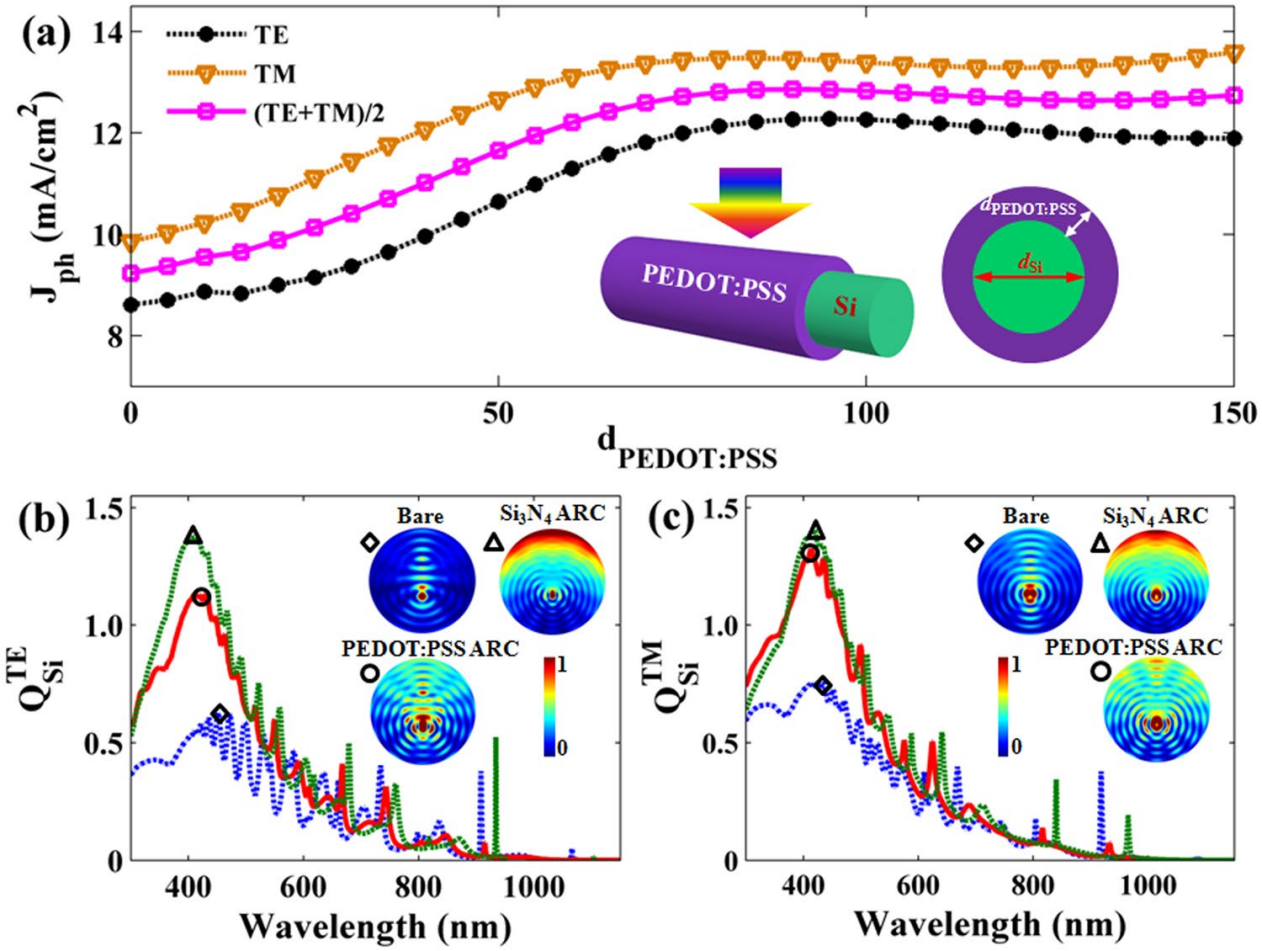
Optical current-density versus thickness of PEDOT:PSS and absorption spectra. (**a**) *J*_ph_ as a function of various *d*_PEDOT:PSS_ for TE, TM and unpolarized incidences under AM1.5G illumination. The insets are 3D and cross-section schematics of PEDOT:PSS SNSC. The *Q*_si_ spectrum of SNSCs with bare, Si_3_N_4_ ARC and PEDOT:PSS ARC under TE (**b**) and TM (**c**) incidences, respectively. The representative cross-sectional absorption patterns of the peaks of curves are also presented in the insets of figures.

The three-dimensional (3D) schematic and cross-section profile of Si/PEDOT:PSS SNSC are shown in the inset of Fig. 1a, where the cylindrical core of crystalline silicon is shell-coated by PEDOT:PSS layer with a uniform thickness denoted as *d*_PEDOT:PSS_. Moreover, according to previous publications^7,10^, the diameter of silicon core (*d*_si_) is fixed at 600 nm. In order to investigate the photoelectrical properties, conventional *p*-*i*-*n* doped homojunction SNSC is adopted for reference, of which the diameter of SiNW is fixed at 600 nm^7^. Thickness of Si_3_N_4_ ARC is chosen as 60 nm in this study, due to the results of optical absorption optimization as shown in Figure S1.

First of all, optical performances of Si/PEDOT:PSS SNSC are shown in Fig. 1. Besides the hole transport layer, PEDOT:PSS works as an outstanding ARC^36,37^. Thus, the thickness of PEDOT:PSS plays an important role in the light-trapping for Si/PEDOT:PSS SNSC. To visually present the influence of PEDOT:PSS thickness on light absorption, we illustrate the dispersion characteristics of light absorption efficiency (*Q*_si_) values of Si/PEDOT:PSS SNSC based on the different *d*_PEDOT:PSS_ under transverse electric (TE, electric field parallel to the axis) and transverse magnetic (TM, magnetic field parallel to the axis) incidences in Figure S2. Moreover, the corresponding photocurrent density (*J*_ph_) curves of Si/PEDOT:PSS SNSCs are displayed in Fig. 1a, yielding the maximum value of 12.69 mA/cm^2^ with an optimal PEDOT:PSS layer (90 nm) under the unpolarized case (*i.e*., 12.12 mA/cm^2^ for TE and 13.26 mA/cm^2^ for TM). Due to the parasitic absorption in PEDOT:PSS layer, there is a tradeoff of the optimized thickness for light absorption of Si core. For further insight of optical absorption, we plot the *Q*_Si_ spectra of SNSCs with the 90 nm PEDOT:PSS layer compared to that of SNSC without ARC or with 60 nm Si_3_N_4_ layer under TE and TM incidences, as shown in Fig. 1b and c. There are no apparent differences in the *Q*_Si_ curves of SiNWs under both TE and TM incidences, which are dominated by light absorption of silicon at short wavelengths (300 nm < λ < 500 nm) and oscillatory decay at long wavelengths (500 nm < λ < 1150 nm). The reasons for this phenomenon are shown as follows: 1) SiNW in a diameter of 600 nm can almost absorb the incident short wavelength light due to the high absorption coefficient of silicon; 2) the absorption ability of SiNW declines with the wavelength increasing due to the degraded light-trapping at long wavelengths; 3) many absorption peaks occur in the long waveband due to the high order optical resonant modes inside the SiNW.

It is obvious that the light absorption abilities of SiNWs with Si_3_N_4_ and PEDOT:PSS ARCs are higher than that of SiNW without ARC under both incidences, especially in the wavelength range of 300–500 nm. Furthermore, the representatives of cross-section absorption patterns in the peaks of *Q*_si_ curves reveal that the optical absorption of core-Si increase significantly by the light management of ARCs for both TE and TM incidences in the insets of Fig. 1b and c. The dazzling hot spots for ARC configurations almost cover the entire structure, due to the strengthened optical antenna and nano-focusing effect by the ARCs. Nano-focusing effect is beneficial to improving the light absorption of Si nanowire, thanks to that it promotes the nanowires to absorb the sideward incident light and focus the inside light at the bottom of nanowire, where the hook face of nanowire looks like a curved lens. For a direct comparison of absorption performances, we summarize the *J*_ph_ values of three related devices in the Table S1. It shows that PEDOT:PSS has a similar light-trapping capability with the conventional Si_3_N_4_ ARC (only less than 1.08 mA/cm^2^ in *J*_ph_), implying that PEDOT:PSS as the eligible ARC is a suitable choice to achieve the outstanding optical performances.

As we know, the photovoltaic performance is also governed by the carrier dynamic processes of generation, transportation, recombination and collection^38,39^. Here, the electrical characteristics of Si/PEDOT:PSS SNSC are carefully examined, primarily focusing on the carrier transport and recombination processes. Among these, bulk recombination can be neglected due to ultra-short carrier diffusion length in this coaxial solar cell, and thus the electrical properties of SNSC depend on the surface recombination and the property of Si/PEDOT:PSS heterojunction^40^. As previously reported, the Si/PEDOT:PSS contact was treated as either Schottky junction where the highly conductive PEDOT:PSS served as the metallic window electrode^41^, or abrupt *p* ^+^ *n* heterojunction where PEDOT:PSS acted as the *p*-type layer^42^, providing charge carrier separation by its hole-selective property. In this study, the *p* ^+^ *n* heterojunction is adopted to implement the carrier dynamic processes (Figure S3), and a strong inversion layer originates from the difference of Fermi energy of Si and work function of PEDOT:PSS. This barrier prohibits the electrons from diffusing into PEDOT:PSS layer, and thus effectively separate photo-generated charge carriers. To thoroughly understand the heterojunction of Si/PEDOT:PSS, the cross-sectional potential distributions under thermal equilibrium as well as the corresponding parameters layouts are illustrated in Figure S4. The built-in electric filed (*V*_bi_) of Si/PEDOT:PSS heterojunction is 854 mV, less than that 971 mV of the *p*-*i*-*n* homojunction. There is a decrement of 117 mV, resulted in a low *V*_oc_ to some extent, if only in consideration of *V*_bi_ values.

We further illustrate the influence of surface recombination velocities on the *J*_sc_ and *V*_oc_ of SNSCs as a basis of the surface recombination velocities (*S*_eff_). The *J*_sc_ is obtained by integrating the external quantum efficiency (*EQE*) spectrum^41^:

1 $${J}_{{\rm{sc}}}=q{\int }_{300}^{1150}{F}_{{\rm{S}}}(\lambda )EQE(\lambda ){\rm{d}}\lambda $$

2 $$EQE(\lambda )={j}_{S}(\lambda )/q{b}_{S}(\lambda )$$

3 $${j}_{{\rm{s}}}(\lambda )=\frac{q\iiint G(\lambda ){\rm{dV}}}{\iint dS}\,-\,\frac{q\iiint {{\rm{U}}}_{{\rm{bulk}}}(\lambda ){\rm{dV}}}{\iint dS}\,-\,\frac{\iint {{\rm{J}}}_{{\rm{surf}}}(\lambda ){\rm{dS}}}{\iint dS}$$

where *EQE* is the ratio of the number of charge carriers collected by solar cell to incident photons, *j*_s_ is the frequency dependent photocurrent density contributed from effective carrier, *b*_s_ is the solar incident photon flux spectrum (AM1.5G), *U*_bulk_ is the internal recombination rate, *U*_surf_ is the surface recombination rate, *V* is the volume of Si layer, and *S* is the surface area of cell. Here, the bulk recombination from radiative, Auger and Shockley-Read-Hall recombination is neglected due to the ultra-short diffusion length in this coaxial device. Owing to the silicon dangling bonds at the surface and the disturbed crystal lattice, a large density of defects within the bandgap exist at the surface and thus interface recombination is the main power loss in the SNSC. The current density loss of surface recombination (*J*_surf_) is expressed as the following equation^43^:

4 $${J}_{{\rm{surf}}}=q{\rm{\delta }}p{S}_{{\rm{eff}}}$$

where δ*p* is the excess minority carrier concentration at the surface. Therefore, the *J*_sc_ of Si/PEDOT:PSS SNSC is determined by the minority carrier concentration of the interface as well as the surface recombination velocity under the constant carrier generation. Furthermore, *V*_oc_ can be expressed by the following equation:

5 $${V}_{{\rm{oc}}}=\frac{kT}{q}\,{\rm{ln}}(\frac{{J}_{{\rm{sc}}}}{{J}_{0}}+1)$$

where *J*_0_ is the dark saturation current density.

**Figure 2:**
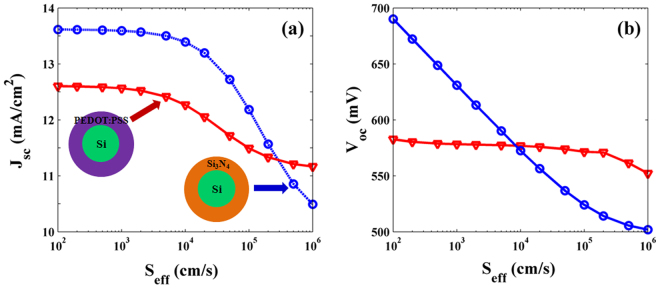
*J*_sc_ and *V*_oc_ versus surface recombination velocity. (**a**) *J*_sc_ and (**b**) *V*_oc_ of Si/PEDOT:PSS and Si/Si_3_N_4_ SNSCs as a function of surface recombination velocity (*S*_eff_).

Figure 2 shows the relationship between *J*_sc_, *V*_oc_ and *S*_eff_ of SNSCs. It is obvious that *J*_sc_ values almost remain steady between 10^2^ cm/s and 10^3^ cm/s, and then a considerable decrease occurs from 10^3^ cm/s to 10^6^ cm/s in Fig. 2a. *J*_sc_ decreases from 12.60 (13.62) mA/cm^2^ to 11.16 (10.49) mA/cm^2^ with the decrement of 1.44 and 3.13 mA/cm^2^ for Si/PEDOT:PSS and Si/Si_3_N_4_ SNSCs, respectively. However, the decrement rate of Si/PEDOT:PSS SNSC is much smaller than that of Si/Si_3_N_4_ SNSC so that the *J*_sc_ values of Si/PEDOT:PSS SNSCs are larger than that of Si/Si_3_N_4_ SNSCs when *S*_eff_ > 5 × 10^5^ cm/s. Figure 2b clearly reveals that with the increase of *S*_eff_, *V*_oc_ of Si/PEDOT:PSS SNSC can be well maintained, while that of Si/Si_3_N_4_ SNSC decrease rapidly. Evidently, there is a sharp decrease of 188 mV for Si/Si_3_N_4_ SNSC, from 690 mV (*S*_eff_ = 10^2^ cm/s) to 502 mV (*S*_eff_ = 10^6^ cm/s), whereas there is not much difference in the *V*_oc_ for Si/PEDOT:PSS SNSC between 582 mV (*S*_eff_ = 10^2^ cm/s) and 552 mV (*S*_eff_ = 10^6^ cm/s). Furthermore, the apparent difference of 108 mV between Si/PEDOT:PSS and Si/Si_3_N_4_ SNSCs in the *V*_oc_ is presented when surfaces are well passivated (*S*_eff_ = 10^2^ cm/s), due to the different built-in potential as shown in Figure S4. It is worth noting that when *S*_eff_ exceeding the turning point of *V*_oc_ curves (10^4^ cm/s), the Si/PEDOT:PSS SNSC displays larger *V*_oc_ values compared to that of Si/Si_3_N_4_ SNSC. Consequently, although the *ψ* value of coaxial Si/PEDOT:PSS heterojunction is not high enough, SNSC still show excellent photovoltaic performances even at high *S*_eff_, being the sufficient tolerance to the surface recombination.

**Figure 3:**
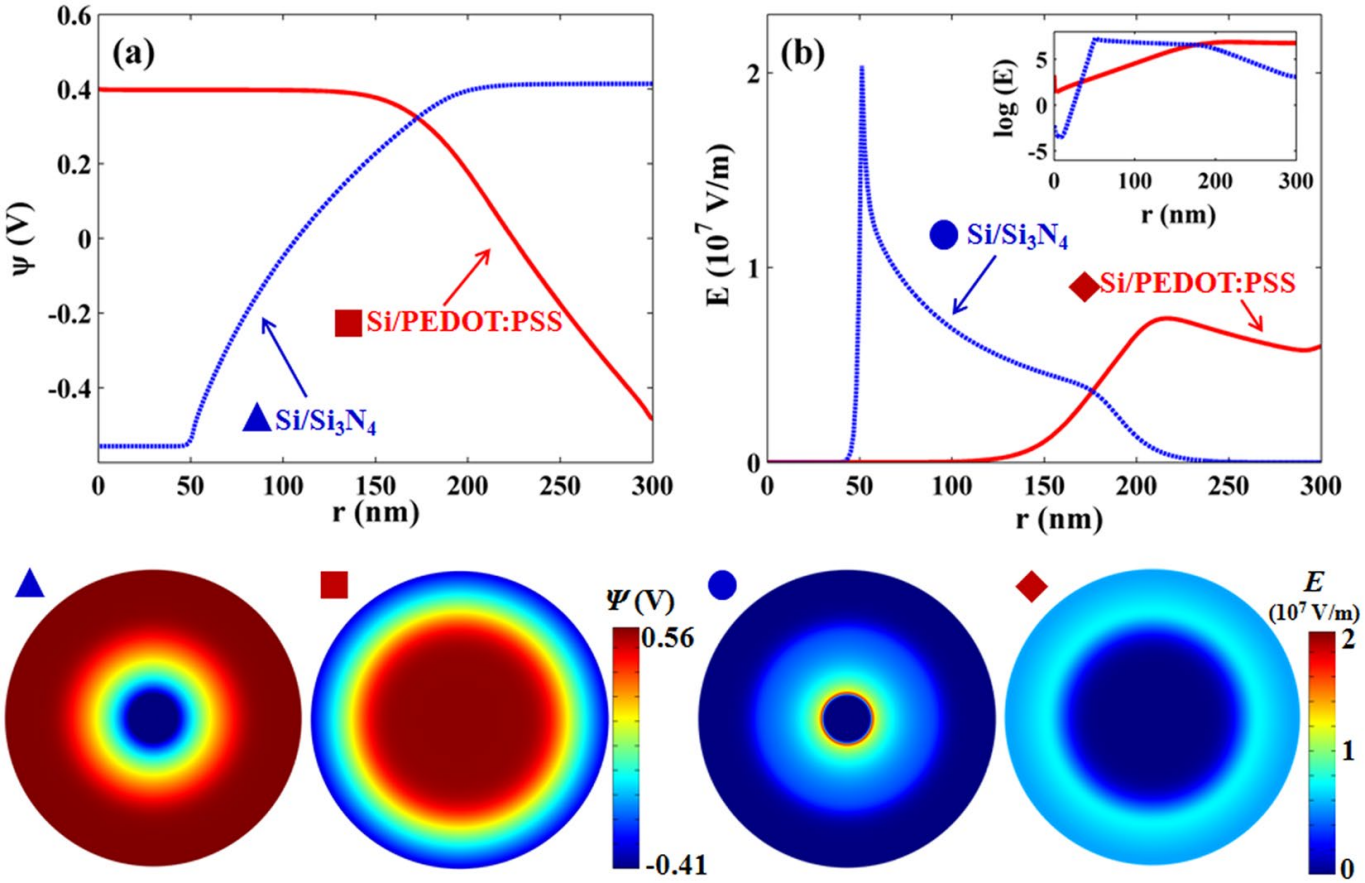
Potential and electric field distribution. (**a**) potential and (**b**) electric field strength along the radial direction for homojunction and heterojunction SNSCs under 600 nm light illumination. The corresponding 2D images are at the bottom of the figure.

We further examine the potential (*ψ*) and electrical field strength (*E*) distribution along the radical direction for Si/PEDOT:PSS and Si/Si_3_N_4_ SNSCs under 600 nm light illumination (*S*_eff_ = 10^2^ cm/s), as shown in Fig. 3. Figure 3a clearly shows that the depletion layer of Si/PEDOT:PSS SNSC (belongs to *p* ^+^ *n* heterojunction) distributes along the radial direction, with lower potential at the outer shell. The minority carrier concentration at the depletion layer cannot be affected by the surface defects, depending on the *n*/*p* doping levels and electrical field passivation. On the other hand, the depletion of Si/Si_3_N_4_ SNSC (belongs to *p*-*i*-*n* doped homojunction) is located at the intrinsic layer, gentler than that under thermal equilibrium. Figure 3b reveals that higher *E* values at the outer shell of PEDOT:PSS drive the carriers though silicon surface quickly to the PEDOT:PSS layer, not being trapped in the silicon surface defects. Therefore, the outer surface recombination results in little photocurrent leakage, nearly without the influence on the photovoltaic performances.

**Figure 4:**
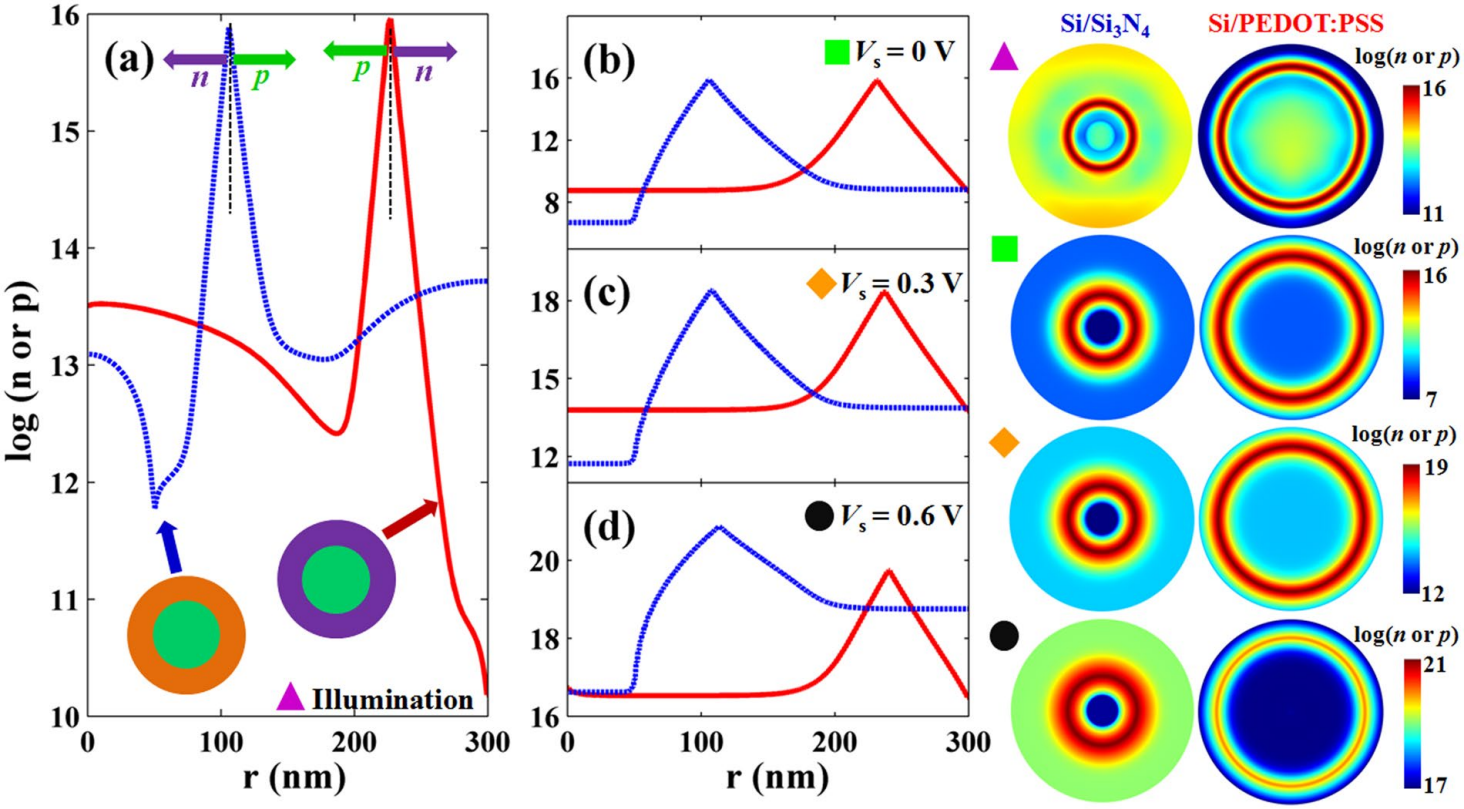
Minority carrier concentrations distribution under different bias voltage. Profiles of minority carrier concentrations along the radial direction under (**a**) light illumination at λ = 600 nm, (**b**) bias voltage *V*_s_ = 0 V, (**c**) 0.3 V, and (**d**) 0.6 V for Si/Si_3_N_4_ and Si/PEDOT:PSS SNSCs. The corresponding 2D images are in the right position of the figure.

For further insight into the influence of surface recombination on the interfacial property and performances of SNSC, we examine the minority carrier concentrations at Si/PEDOT:PSS and Si/Si_3_N_4_ SNSCs under light illumination and different bias voltages in Fig. 4. Minority carrier concentrations is dependent on the interfacial passivation and junction property. It plays a key role in determining the device outputs. Minority and majority charge carriers locate the opposite side of the device, thus holes (*p*) for Si/Si_3_N_4_ SNSC and electrons (*n*) for Si/PEDOT:PSS SNSC are located at the outer shell of SiNW. Additionally, the peak concentration of minority charge carriers presents at the depletion regions of the junction. The wide depletion region of this kind of nanowire does not affect the carrier diffusion to some extent. However, for the Si/PEDOT:PSS SNSC, the electron concentration at the external shallow surface is much lower than that of Si/Si_3_N_4_ SNSC, leading to less recombination loss at the Si/PEDOT:PSS interface. This phenomenon can be explained that the strong electric field at the surface of Si/PEDOT:PSS heterojunction remarkably reduces the electron concentration to suppress the surface recombination, which is the essential difference between Si/Si_3_N_4_ and Si/PEDOT:PSS SNSCs.

In order to analyze the reinforced electric-field passivation to *V*_oc_ of Si/PEDOT:PSS SNSC, the distributions of minority carrier concentration under different bias voltages (0 V, 0.3 V and 0.6 V) are investigated in Fig. 4b–d. Obviously, there is no apparent difference in the minority carrier of outer-sided interface under *V*_s_ = 0 and 0.3 V, showing the similar recombination levels. Notably, when *V*_s_ = 0.6 V, the concentration of minority carrier at outer-sided surface of Si/Si_3_N_4_ system is far beyond that of Si/PEDOT:PSS one, resulting in serious surface recombination and degraded *J*_sc_ and *V*_oc_. Therefore, the Si/PEDOT:PSS SNSC is beneficial for the electrical performances, compared to the conventional Si/Si_3_N_4_ SNSC.

**Figure 5:**
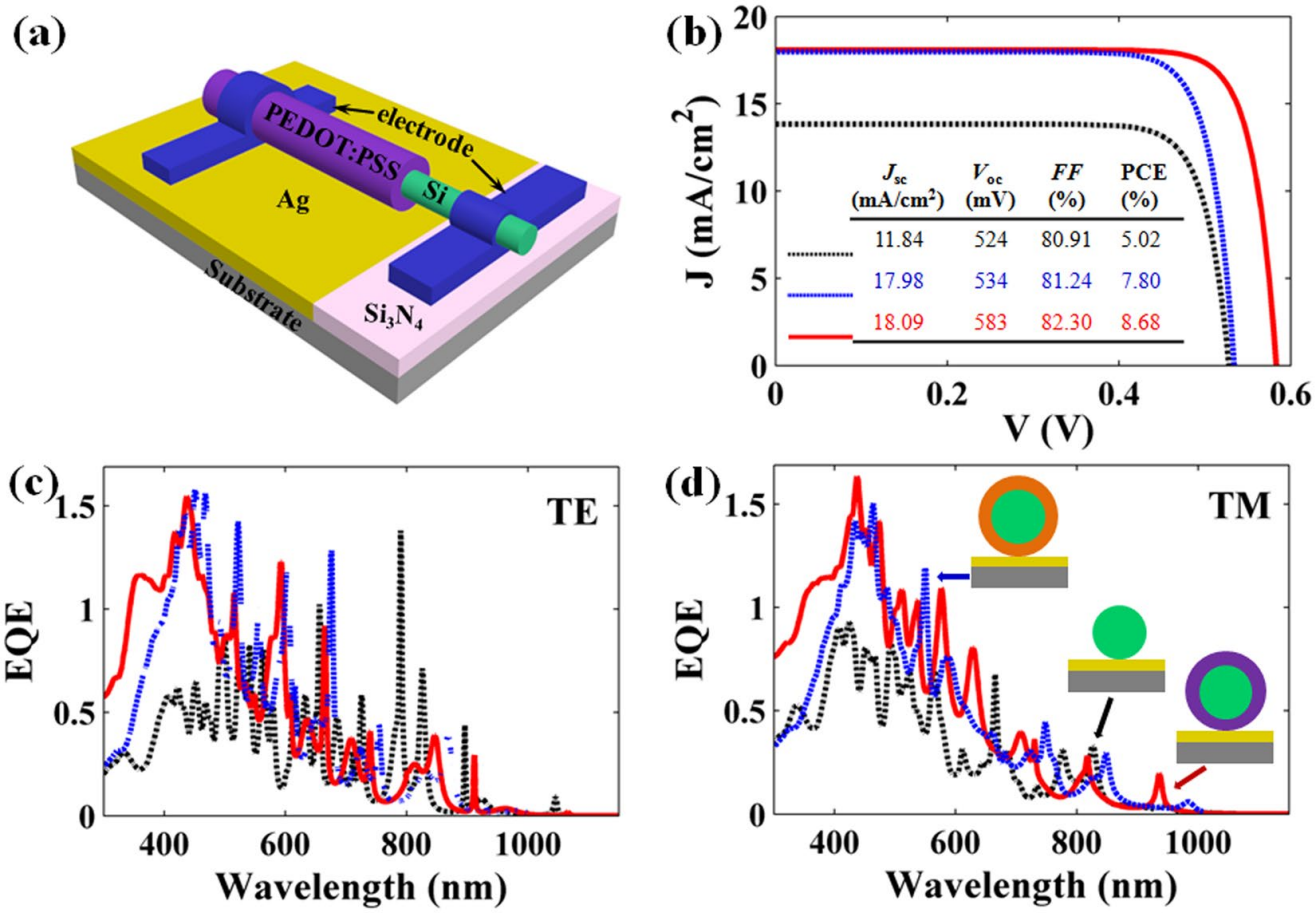
Schematic diagram with substrate as well as *EQE* and *I-V* characteristic curves. (**a**) Schematic diagram of Si/PEDOT:PSS heterojunction SNSC with an Ag back reflector on the substrate, (**b**) *J*-*V* curves, and *EQE* spectra under (**c**) TE and (**d**) TM incidences of bare, Si_3_N_4_ and PEDOT:PSS SNSCs.

As above discussions, we consider the SiNW as a freestanding setup, which is hard to implement in reality. Considering the fabrication feasibility, we would like to have an extended discussion on the SNSCs lying on a metal-coated substrate. Here, the optimized parameters (i.e., *d*_Si_ = 600 nm and *d*_PEDOT:PSS_ = 90 nm) for Si/PEDOT:PSS SNSC are chosen and a 200 nm silver film is deposited on the substrate as a back reflector for further improving the light-harvesting in Fig. 5a. Here, to mimic the carrier transport process, the surface recombination velocity of double systems was fixed at 10^5^ cm/s (a suitable value for nanowire). By addressing the accurate photoelectrical simulations, the corresponding current density-voltage (*J-V*) curves of SNSCs are displayed in Fig. 5b. Inspiringly, the Si/PEDOT:PSS SNSC yields the best PCE of 8.68%, with a *J*_sc_ of 18.09 mA/cm^2^, a *V*_oc_ of 583 mV and a fill factor (*FF*) of 82.30%, an enhancement of 72.91% (21.13%) from that of the bare (Si_3_N_4_) homojunction SNSC. The improved PCE can be ascribed to the modified *J*_sc_ (*i.e*., from 11.84 and 17.98 mA/cm^2^ of bare and Si_3_N_4_ SNSCs to18.09 mA/cm^2^ of PEDOT:PSS SNSC) and enhanced *V*_oc_ (*i.e*., from 524 and 534 mV of bare and Si_3_N_4_ SNSCs to 583 mV of PEDOT:PSS SNSCs) due to the suppressed surface recombination. In order to take *J*_sc_ enhancement into consideration, we carefully examine the *EQE* spectra of SNSCs for TE and TM incidences in Fig. 5c and d, respectively. At first glance, the *EQE* values are remarkably enhanced with the presence of rear metallic reflector for both incidences compared with the *Q*_Si_ spectra of Fig. 1. Particularly in the short wavelength region, the metal layer reflects the unabsorbed short-wavelength light back into the SiNW cavity for the second-round absorption, leading to the enhanced optical absorption as well as improved *EQE*. As a result, *J*_ph_ values can be as high as 13.59 (13.10), 20.49 (19.75) and 18.33 (19.53) mA/cm^2^ for bare, Si_3_N_4_ and PEDOT:PSS SNSCs under TE (TM) incidences, respectively, significantly higher than that of the free standing devices (summarized in Table S1 in the Supporting Information). Since the detailed electrical simulations reveal that the Si/PEDOT:PSS SNSC has better properties in the electrical dynamic processes (carrier collection and recombination), compared to that of the homojunction systems. The *J*_sc_ values of SNSCs are 11.84, 17.98 and 18.09 mA/cm^2^ for bare, Si_3_N_4_ and PEDOT:PSS ARCs under unpolarized case, with the current density loss (*J*_ph_ – *J*_sc_) of 1.51, 2.14 and 0.84 mA/cm^2^, respectively. Thus, the Si/PEDOT:PSS SNSC is more suitable for nanodevices for light-harvesting to yield a large *J*_sc_ value. In a word, the Si/PEDOT:PSS SNSC has the outstanding optical and electrical performance with Ag reflector, as a feasible alternative to achieve high efficiency SNSCs.

In addition, we briefly study the stabilized spatial profiles of electron/hole concentration and electrostatic potential (*n*, *p* and *ψ*) under 600 nm light illumination for the Si_3_N_4_ and PEDOT:PSS systems as shown in Figure S5. It is obvious that *n* and *p* profiles of the two SNSCs are primarily dependent on the initial doping conditions. Although the photo-generated charge carriers play a key role in the power output of SNSCs, the *n* and *p* concentrations are slightly modified, which are too little to change the profiles of carrier concentration distribution^44^. And thus, the corresponding *ψ* profiles of SNSCs under illumination case display the similar distribution as these of the thermal equilibrium in the dark, ensuring a large *V*_bi_ and *V*_oc_.

## Discussion

In summary, we propose a high efficiency heterojunction SNSC by combining the *n*-SiNW with *p*-type PEDOT:PSS compared to the conventional *p*-*i*-*n* homojunction SNSC. This structure serves as a potential alternative for simple manufacturing process in power nanodevice. The PEDOT:PSS ARC with optimized thickness of 90 nm efficiently absorb visible light, leading to 42.65% increase of photocurrent, due to nano-focusing effect, especially for short-wavelength light. In addition, larger junction area at hollow *p* ^+^ *n* heterojunction is beneficial for the photo-generated carrier separation, compared to that at homojunction. The photovoltaic properties (*J*_sc_ and *V*_oc_) of PEDOT:PSS SNSC can be well maintained even in large surface recombination velocity, due to the abrupt *p* ^+^ strong inversion layer and field-effect passivation contributes to suppress the surface recombination. Furthermore, with an Ag back reflector, a high PCE of 8.68% of PEDOT:PSS SNSC is predicted, with an enhancement of 72.91% and 11.13% from that of the bare and Si_3_N_4_ SNSCs, displaying its superior power supply capability in various nanodevices.

## Methods

### Simulation methods

In this study, we implement the optical simulations by solving Maxwell’s equations, based on the finite element methods. The length of axial SiNW is assumed to be far beyond its diameter, enabling the use of the two-dimensional (2D) model^11^. The wavelength range of 300–1150 nm is considered to fit the light response of SNSC and absorption bandgap of Si. Based on the optical constants from Palik table^45^, we systematically evaluate the optical performances, including the *Q*_si_ and the steady distribution of electromagnetic field. In this study, *Q*_Si_ is defined as the absorption cross section (*C*_abs_) divided by the geometric cross section (*C*_geom_), i.e., *Q*_Si_ = *C*_abs_/*C*_geom_. Here, *C*_geom_ is defined as the product of the diameter and the length of nanowire, which can be reduced to the value of diameter in two dimensional models due to the ultra-high length/diameter ratio of nanowire structures. While, the actual light absorption area of nanowire is greater than the physical size due to the optical antenna effect, leading to that the maximal value of *Q*_Si_ beyond 100% is possible. In terms of typical TE and TM polarizations of optical wave, the unpolarized incidence is regarded as the average of TE and TM incidences^10,11^. Quantifying the overall optical performance, the *J*_ph_ is calculated by integrating the absorption spectrum of Si core under the standard AM1.5G illumination:

6 $${J}_{ph}=q{\int }_{300}^{1150}{F}_{S}(\lambda ){Q}_{Si}d\lambda $$

where *q* is the element charge and *F*_S_ is the incident AM1.5G solar spectrum. We mainly conduct the optoelectronic simulations to evaluate the electrical properties of SNSC in the carrier charge transport and recombination processes: stabilized carrier/potential profiles, *EQE* spectra, *J*-*V* characteristics, which is implemented by solving the carrier transport and Poisson’s equations based on the optical generation. The detailed information on the optoelectronic simulation technique can be found in previous publications^46–51^.

### Fabrication possibility

**Figure 6:**
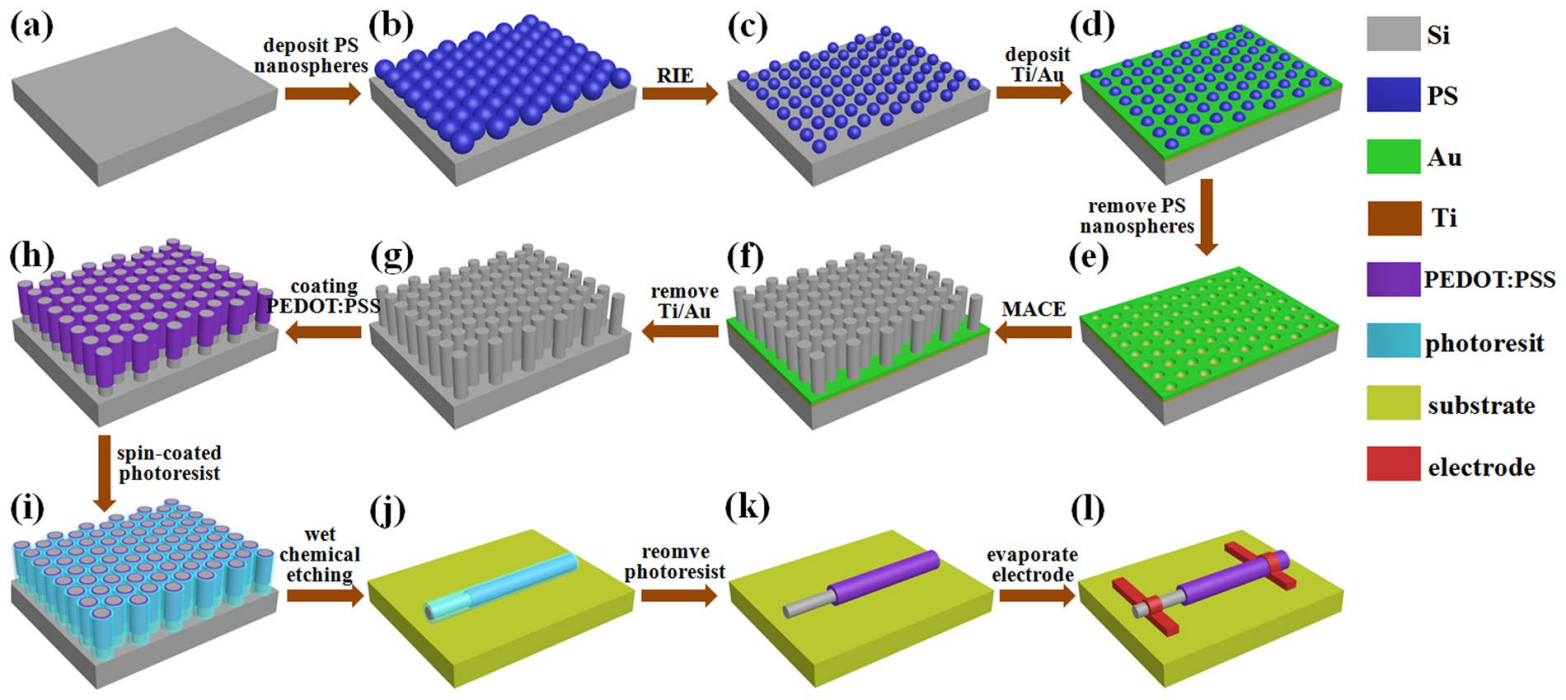
Fabrication procedure. Suggested fabrication procedure of Si/PEDOT:PSS SNSC.

A suggested fabrication process for the PEDOT:PSS SNSC is given as shown in Fig. 6 ^7,12,38^. The SiNWs can be prepared by metal-assisted chemical etching (MACE) referenced in our previous work^52^. Firstly, a monolayer of polystyrene spheres (PS) with diameter of 900 nm is deposited on the polished-side of wafer by a microinjection process, followed by the reactive ion etching (RIE) to get the desired diameter. Then a Ti/Au bilayer is deposited on the PS-modified wafer with the thicknesses of 2/20 nm by electron beam evaporation. After removing the PS coating by using methylbenzene solution, the wafer is immersed in the HF (49%) and H_2_O_2_ (31%) deioned water solution (10:1:40 in volume ratio) to get the SiNWs in a desired length. The Au mesh is removed by the 5 wt% I_2_ and 10 wt% KI water solution. Secondly, the n-Si with the standing SiNWs are covered by the PEDOT:PSS layer *via* the vapor phase polymerization method to form the coaxial heterojunction. Then a photoresist layer is spin-coated on the PEDOT:PSS layer as the protective layer during the silicon wet chemical etching (KOH etchant)^7^. Finally, the standard electron beam lithography and thermal evaporation are used to fabricate coaxial Si/PEDOT:PSS nanowire devices, with selective contacts on the *n*-core Si and *p*-shell PEDOT:PSS.

## Electronic supplementary material

Supplementary information
