# Supplementary material for: Opto-electric investigation for Si/organic heterojunction single-nanowire solar cells
Source: Sci Rep. 2017 Nov 6;7:14575. doi: 10.1038/s41598-017-15300-0 (PMC5674000; doi:10.1038/s41598-017-15300-0)
Supplement: Supplementary file 1 — Supplementary information [file 41598_2017_15300_MOESM1_ESM.docx]

**Opto-electric investigation for Si/organic heterojunction single-nanowire solar cells**

**Zhenhai Yang^1,#^, Zhaolang Liu^1,2,#^, Jiang Sheng^1,*^, Wei Guo^1^, Yuheng Zeng^1^, Pingqi Gao^1^, and Jichun Ye^1,*^**

^1^Ningbo Institute of Material Technology and Engineering, Chinese Academy of Sciences, Ningbo 315201, China

^2^School of Materials Science and Engineering, Shanghai University, Shanghai, 200072, China

*Correspondence authors: shengjiang@nimte.ac.cn (JS); jichun.ye@nimte.ac.cn (JY)

#Equal contributors

*
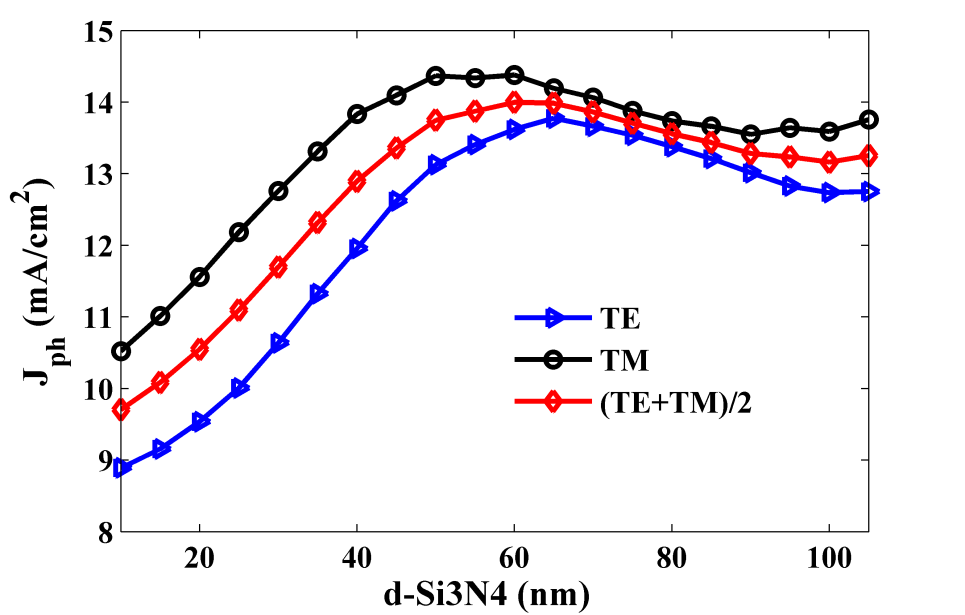
*

**Figure S1** *J*_ph_ as a function of thicknesses of Si_3_N_4_ under TE, TM and unpolarized incidences.

**
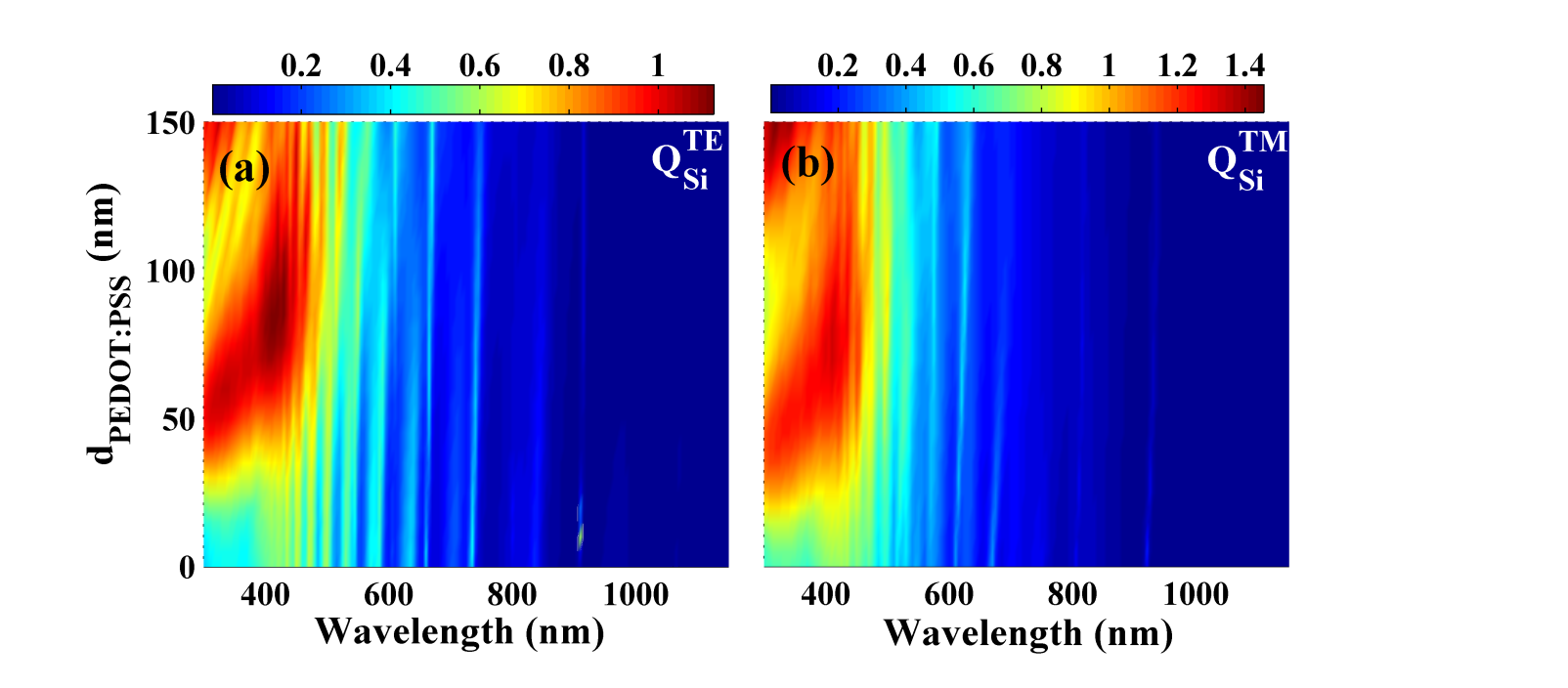
**

**Figure S2** Absorption efficiencies as the basis of the light wavelength and PEDOT:PSS thickness (*d*_PEDOT:PSS_) under TE (a) and TM (b) incidences.

**Table S1** *J*_ph_ and *J*_sc_ of NWs with bare, Si_3_N_4_ ARC, PEDOT:PSS ARCs under TE, TM and unpolarized incidences for the wo (without substrate) and sub (with rear Ag substrate) configurations.

**
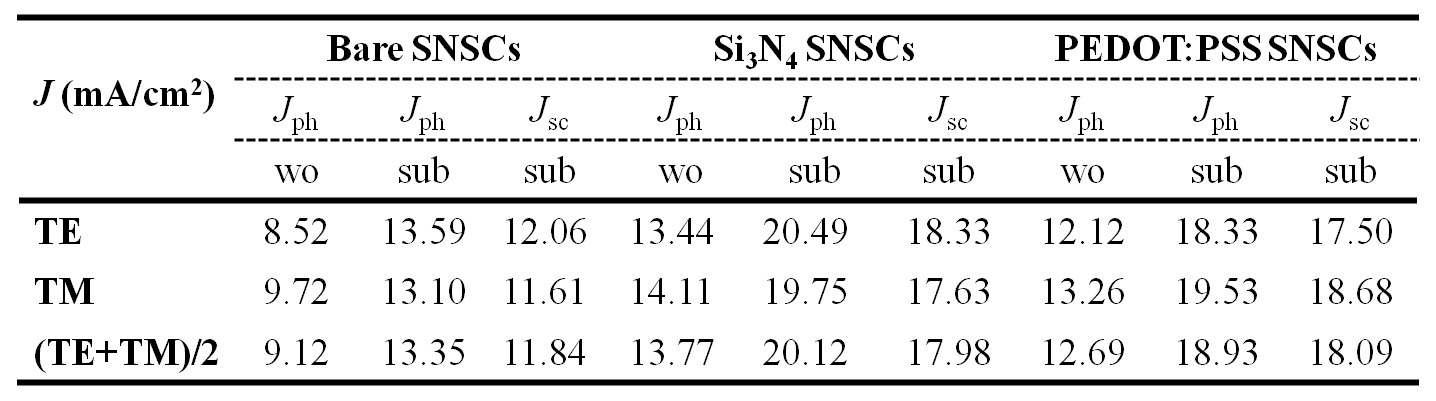
**

**
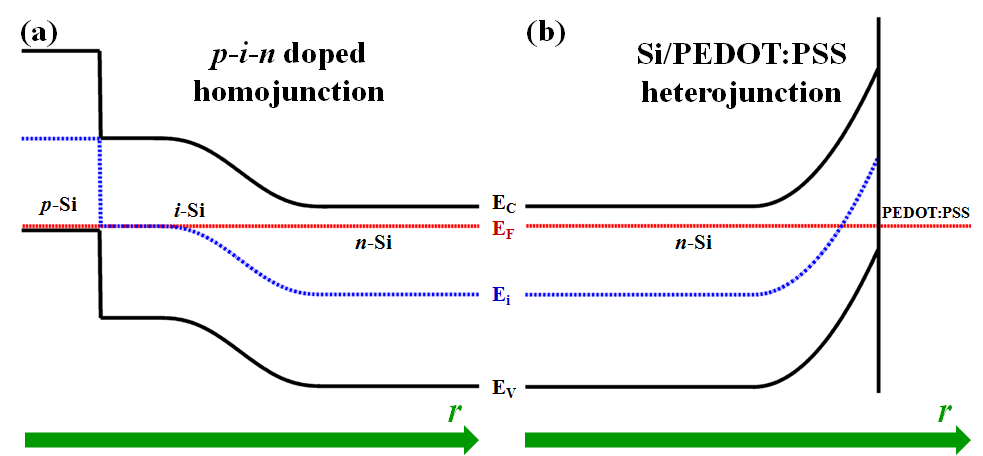
**

**Figure S3** Schematic of the energy band structures for (a) the conventional *p*-*i*-*n* homojunction and (b) Si/PEDOT:PSS heterojunction devices.

**
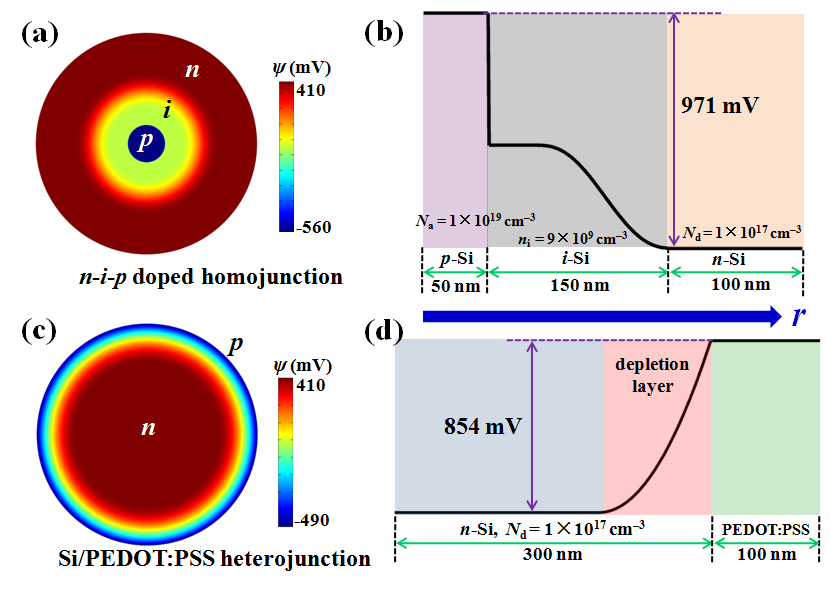
**

**Figure S****4** Potential distributions of cross-sectional profiles under thermal equilibrium for (a) *p*-*i*-*n* doped homojunction NW and (c) Si/PEDOT:PSS heterojunction NW, (b) and (d) the corresponding electrical parameters including the potential line profile and doping concentrations.


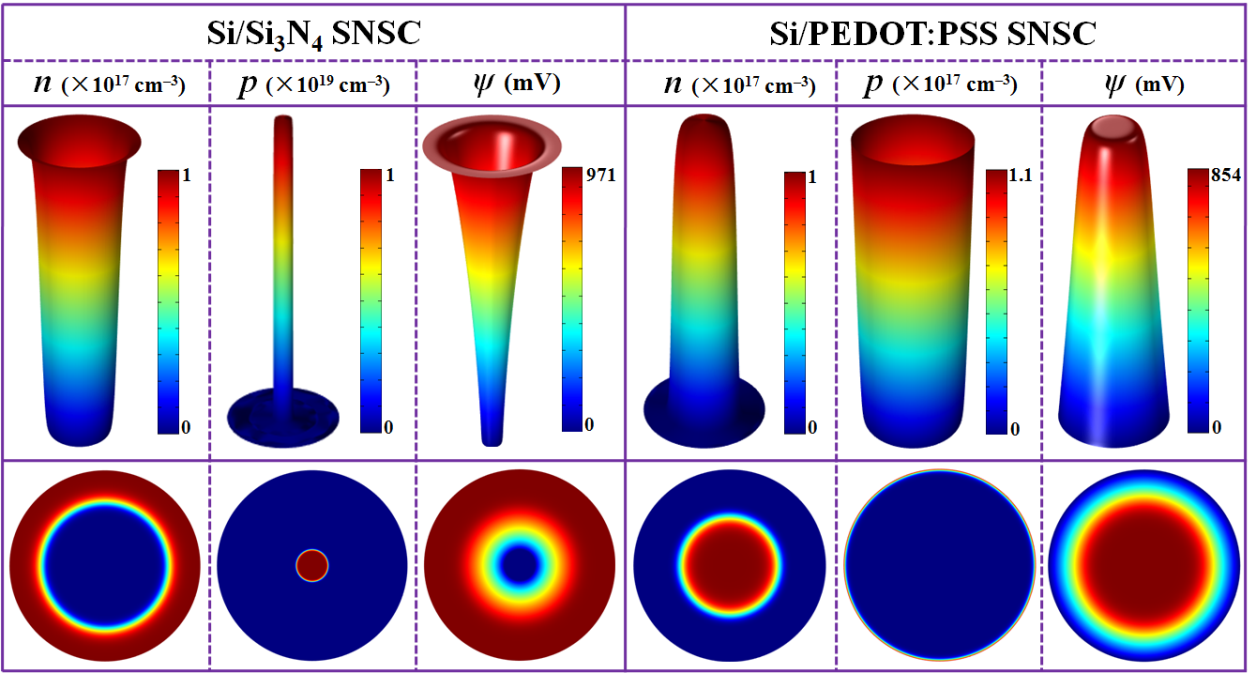


**Figure S5** Stabilized spatial distributions of *n, p* and *ψ* in Si_3_N_4_ and PEDOT:PSS SNSCs under light illumination at λ = 600 nm, the corresponding two-dimensional images are in the second row.
